# Extracorporeal membrane oxygenation for acute pulmonary embolism after postoperative craniocerebral trauma: a case report

**DOI:** 10.3389/fcvm.2023.1200553

**Published:** 2023-06-08

**Authors:** Xiaozu Liao, Xiaojuan Chen, Shi Zhong, Junlin Wen, Binfei Li

**Affiliations:** ^1^ Department of Anesthesiology, Zhongshan City People’s Hospital, Zhongshan, China; ^2^ Department of Anesthesiology ICU, Guangzhou Panyu Central Hospital, Guangzhou, China

**Keywords:** craniocerebral trauma, acute pulmonary embolism, VA-ECMO, anticoagulation, bleeding

## Abstract

**Introduction:**

Massive pulmonary embolism (PE) is a life-threatening complication of major surgery with a mortality rate of up to 50%. Extracorporeal membrane oxygenation (ECMO) is primarily used for respiratory and circulatory support. Venoarterial extracorporeal membrane oxygenation (VA-ECMO) is used to stabilize patients with acute massive PE. Acute brain injury, vascular disease, and immunosuppression are contraindications to ECMO, as stated in the 2021 Extracorporeal Life Support Organization guidelines.

**Case summary:**

We report a case of a patient with craniocerebral trauma whose postoperative course was complicated by massive PE and subsequent cardiac arrest that required urgent VA-ECMO, followed by anticoagulation with heparin. The patient showed hemodynamic improvement and was discharged 68 days after hospitalization.

**Discussion:**

ECMO has gradually been accepted for patients with craniocerebral injuries. The safety and effectiveness of ECMO in patients with craniocerebral injury, along with the optimal duration of ECMO and anticoagulation strategies, require further study.

## Introduction

Venous thromboembolism (VTE), which includes deep vein thrombosis and pulmonary embolism (PE), is the third most common acute cardiovascular syndrome worldwide, after myocardial infarction and stroke (1). PE is the general term for a group of diseases or clinical syndromes in which emboli block the pulmonary artery or its branches, including pulmonary thromboembolism (PTE), fat embolism syndrome, amniotic fluid embolism, air embolism, and tumor embolism. PTE is the most common type of PE (2). High-risk PE is associated with a high early mortality rate (approximately 25%), and up to 65% of patients require cardiopulmonary resuscitation (CPR) (1, 3). Extracorporeal membrane oxygenation (ECMO) is mainly used for respiratory and circulatory support in critically ill patients. Acute brain injury, vascular disease, and immunosuppression are contraindications to ECMO, as stated in the 2021 Extracorporeal Life Support Organization guidelines. Anticoagulation is a common treatment for pulmonary embolism, causing a risk of bleeding especially in patients with acute brain trauma. In this report, we describe the successful application of VA-ECMO for the treatment of cardiac and respiratory arrest caused by acute PE after postoperative craniocerebral trauma.

## Case introduction

**Figure 1:**
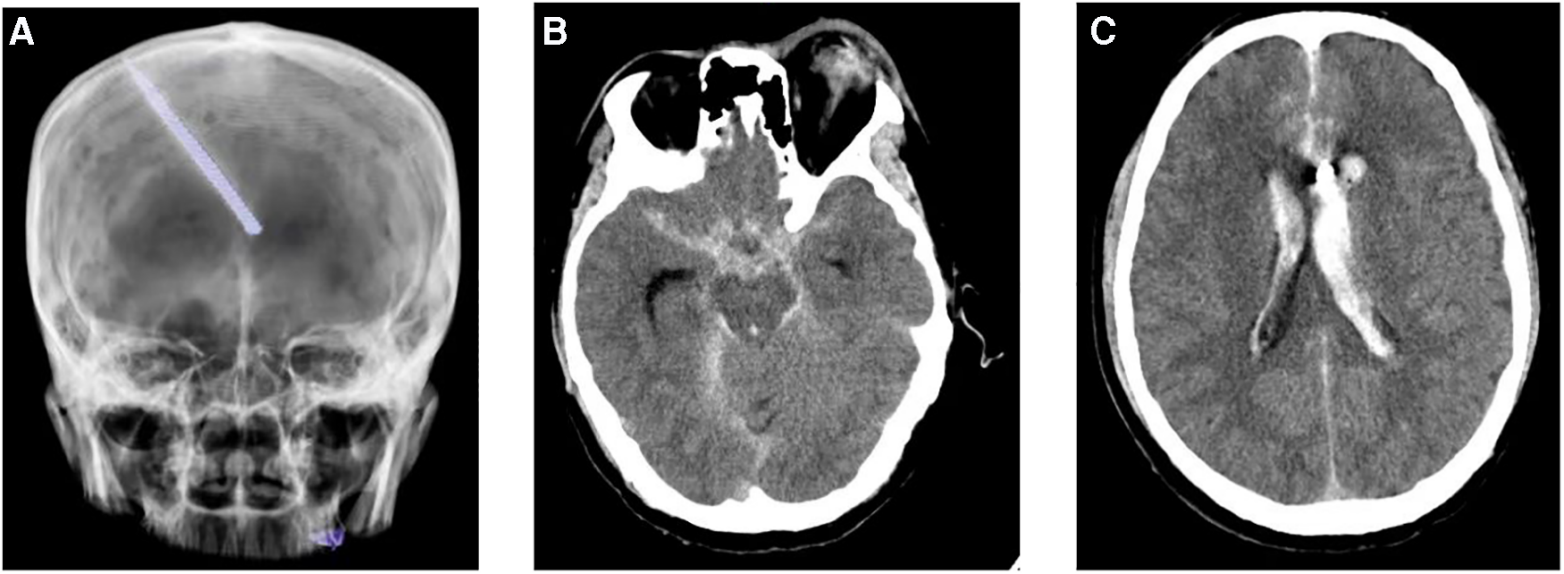
Computed tomography reveals a foreign body (metal strip) spanning the genu of the corpus callosum to the right parietal lobe (**A**); rupture of the left eyeball, subcutaneous hematoma of the eyelid, fracture of the top wall of the left orbit, and a small accumulation of gas in the left orbit (**B**); and contusion of the left frontal lobe with extensive subarachnoid hemorrhage (**C**).

**Figure 2:**
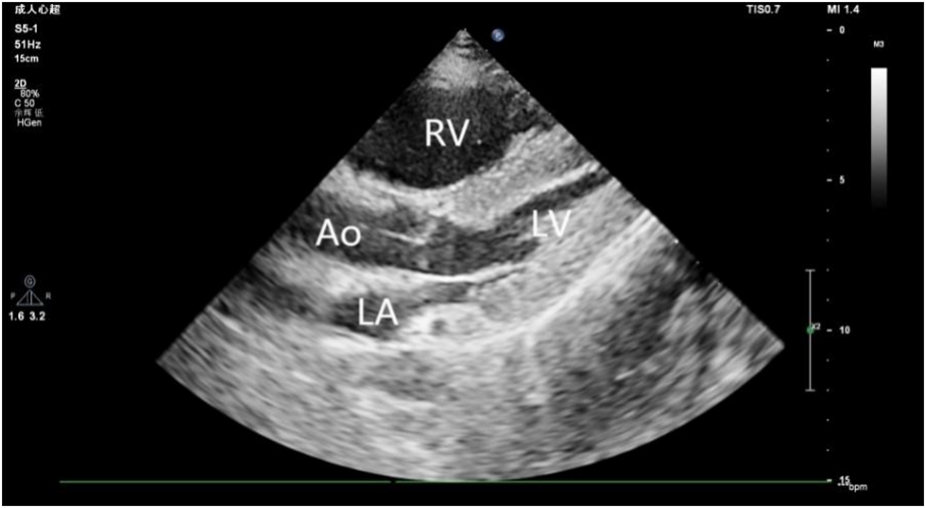
Ultrasound image reveals an enlarged right ventricle and underfilled left ventricle. RV, right ventricle; LV, left ventricle; LA, Left atrium; AO, Aorta.

**Figure 3:**
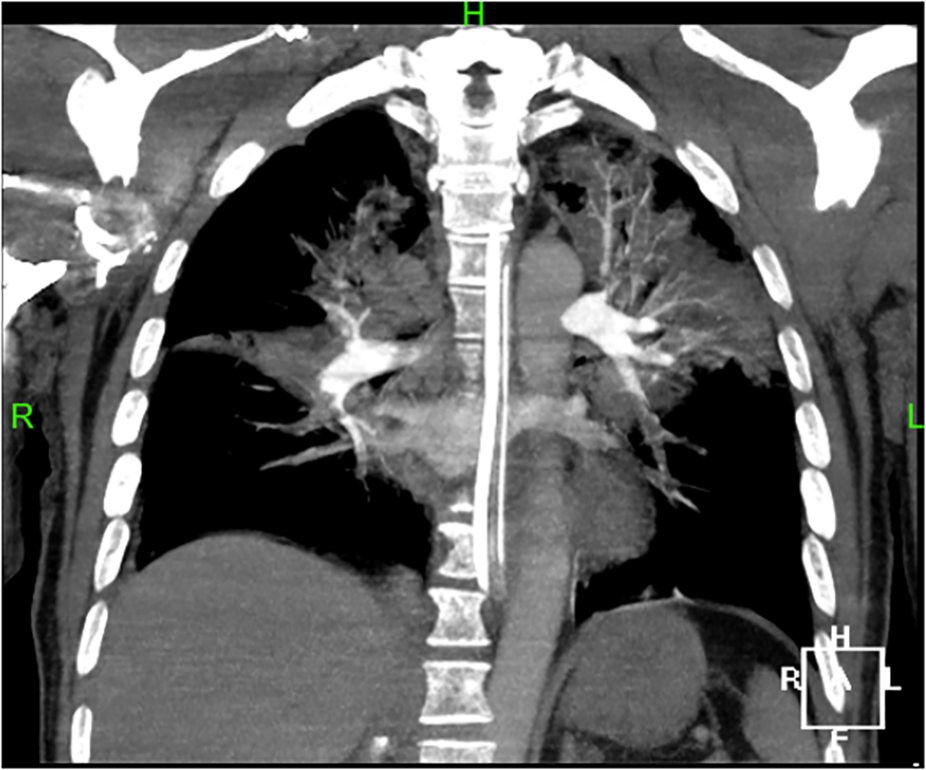
Pulmonary artery CTA shows bilateral lower pulmonary embolism, involving multiple segmental branches.

A 44-year-old male patient experienced bleeding in the left eye and head pain following a nail gun shot to the skull, entering through the left eye on September 26, 2022. Emergency computed tomography (CT) of the brain revealed the presence of a foreign body (metal strip) spanning the genu of the corpus callosum to the right parietal lobe, contusion of the left frontal lobe with extensive subarachnoid hemorrhage, rupture of the left eyeball, subcutaneous hematoma of the eyelid, fracture of the top wall of the left orbit, and a small accumulation of gas in the left orbit (Figure 1). Emergency debridement of the brain with the patient under general anesthesia, removal of the foreign body via brain incision, external ventricular drainage, and implantation of an intracranial pressure sensor were performed. On the 12th postoperative day (October 7), the patient suddenly became unresponsive, the arterial pulse disappeared, and cardiorespiratory arrest occurred. Emergency management was immediately performed, including external chest compressions, endotracheal intubation, ventilator-assisted ventilation, adrenaline injection, and acid correction. The patient did not regain spontaneous circulation or breathing; thus, VA-ECMO support was immediately provided. The left femoral artery and vein were accessed. Because of the high risk of potential bleeding following craniocerebral trauma, the medical team decided not to administer the initial dose of heparin. Instead, they inserted a 17 Fr (depth of 13 cm) and a 21 Fr (depth of 45 cm) Medtronic heparin-coated catheter through the femoral artery and vein, respectively, to facilitate VA-ECMO. The ECMO flow rate was approximately 2.5 L/min, and the blood temperature was maintained at 36°C. Arterial blood gas analysis for emergency examination showed the following: FIO_2_, 100%; pH, 7.28; PCO_2_, 79 mmHg; PO_2,_ 174 mmHg; glucose, 17.30 mmol/L; lactic acid, 14.10 mmol/L; HCO_3_, 32.1 mmol/L; and hemoglobin (Hgb), 74 g/L. The D-dimer level of the patient was 148.68 mg/L, and the hypersensitive troponin T level was 424 ng/L. Bedside color Doppler ultrasonography of the heart indicated an enlarged right ventricle and underfilled left ventricle, suggesting the possibility of PE (Figure 2). Pulmonary artery computed tomography angiography (CTA) revealed the following: (1) bilateral lower PE involving multiple segmental branches and (2) multiple flaky exudations and consolidation foci in both lungs, making it necessary to differentiate between pulmonary infection and pulmonary infarction (Figure 3). Anticoagulation therapy was initiated with a heparin dose of 500 U/h, and ECMO treatment was continued until an activated coagulation time (ACT) of 180–200 s was achieved. Additionally, ventilator-assisted ventilation, anti-infection, prone ventilation, nutritional support, and other treatment were provided, after which the vital signs, blood gas results, lactic acid levels, and other indicators of the patient showed improvement. After 48 h of ECMO treatment (October 9), cardiac systolic function returned to normal, and the vital signs of the patient were stable. ECMO was removed on October 10. Trends in the vital signs, ECMO parameters, and laboratory test values of the patient during ECMO treatment are shown in Table 1.

**Table 1:** Trends In the vital signs, extracorporeal membrane oxygenation parameters, and other laboratory test values of the patient.

	Immediately after ECMO treatment	12 h after ECMO treatment	24 h after ECMO treatment	24 h after ECMO treatment	24 h after ECMO treatment	24 h after ECMO treatment	Weaning from ECMO
Heart rate (beats/min)	144	128	97	91	100	86	81
Blood pressure (mmHg)	122/80	97/71	144/96	143/87	139/82	177/88	120/83
Finger pulse oxygen (%)	81	99	99	99	99	99	99
Norepinephrine (ug/kg/min)	1	0.5	0.2	0.1			
Speed of rotation (r/min)	2,849	2,850	2,879	2,880	2,775	2,464	2,215
Flow rate (L/min)	2.44	2.53	1.87	1.85	1.98	1.25	0.98
ACT (S)		178	180	182	182	189	190
Lactic acid (mmol/L)	8.3	4.4	1.5	1	0.8	0.7	0.6
Oxygen partial pressure (mmHg)	63	69	233	176	209	227	233
Arterial partial pressure of carbon dioxide (mmHg)	73	46	47	43	56	41	30

**Figure 4:**
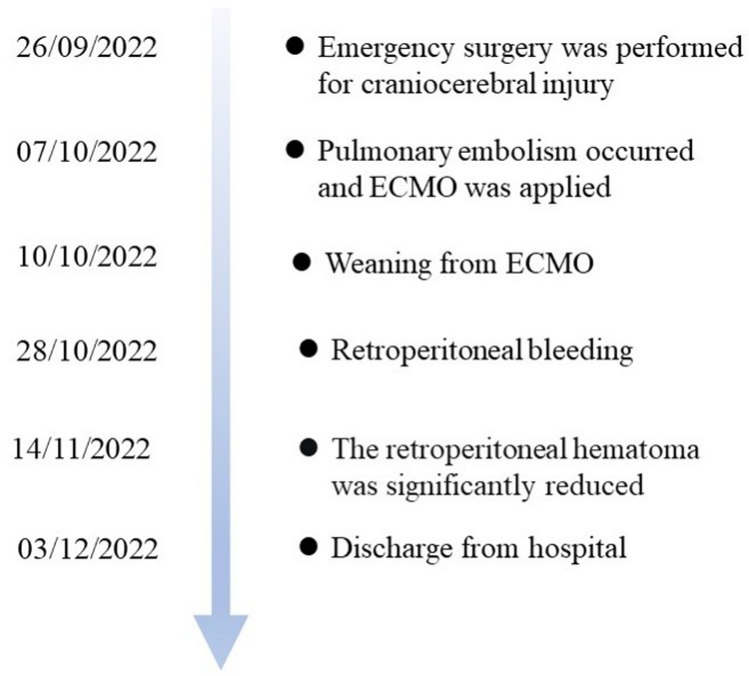
Timeline course for this case.

Following the surgery, heparin was discontinued and later replaced with enoxaparin for anticoagulation therapy (6,000 IU, every 12 h). On October 15, the patient regained consciousness. On October 28, the patient experienced abdominal pain and discomfort, mainly in the right lower abdomen. Physical examination revealed tension and tenderness of the right abdominal muscles, and a blood test showed a Hgb level of 44 g/L. Given the possibility of retroperitoneal bleeding, complete abdominal CT was performed, which revealed the following findings: a thickened right peritoneum, mixed-density right retroperitoneal hematoma measuring approximately 122 mm  ×  84 mm, right iliac muscle hematoma measuring approximately 75 mm  ×  49 mm, and hyperdense strip observed in the pelvis. Enoxaparin was discontinued, drugs were administered to stop the bleeding, and blood transfusion was initiated. However, there was no significant decrease in hemoglobin upon reexamination. On November 14, subsequent abdominal CT showed a decrease in hematoma size. During the follow-up consultation, the patient provided clear responses to questions. He demonstrated left eye blindness and a right eye vision of 0.9. He reported no abdominal pain, and no tenderness was observed. A decrease in the right abdominal mass size was noted. The right limb had a strength level of 5; however, the left upper limb and left lower limb had strength levels of 4 + and 3, respectively. The patient underwent rehabilitation and was successfully discharged 68 days after hospitalization (Figure 4).

## Discussion

Massive PE is a rare but life-threatening form of VTE that poses a great challenge to postoperative patients (4). ECMO is a cardiopulmonary support technique used in patients with severe cardiopulmonary failure. Many studies have reported the use of ECMO as an adjunctive treatment for high-risk PE (5–8). ECMO treatment does not directly affect pulmonary thrombolysis; however, it can offer critical circulatory and respiratory support to patients with a high risk of pulmonary embolism, providing time for patients to receive pulmonary reperfusion therapy or undergo spontaneous thrombolysis. The 2019 European Society of Cardiology and European Respiratory Society guidelines for acute PE state that treatment strategies should be individualized based on risk stratification, with hemodynamically unstable PE classified as a high-risk condition. For high-risk PE, systemic thrombolysis (I/B) is preferred, while surgery (I/C) or percutaneous catheter intervention (IIa/C) are used as alternatives when thrombolysis fails or contraindications are present. The guidelines also suggest that anticoagulation during ECMO therapy as an independent treatment for PE is controversial and recommends that ECMO-supported PE should be combined with surgical thrombectomy or catheter intervention (IIb/C) (1). In a cohort study of 41 patients diagnosed with massive PE between 2015 and 2018, Ghoreishi et al. have found that more than 70% of patients with massive PE who received VA-ECMO support were able to recover with the use of anticoagulation therapy (9). A retrospective study by Liu et al. that included nine patients with PE who received ECMO support has reported that four (44.4%) of the surviving patients received only VA-ECMO and anticoagulant therapy (10). Our patient had high-risk PE following traumatic brain injury and had contraindications to thrombolysis; therefore, no thrombolytic therapy was administered. Considering the result of pulmonary artery CTA that revealed lower PE involving multiple segmental branches, anticoagulant therapy was administered. Surgical or aspiration thrombectomy was considered next-line treatment if anticoagulant therapy did not improve the status of the patient. However, the hemodynamics and oxygenation of the patient improved after anticoagulation therapy, suggesting that anticoagulation therapy alone can be effective.

As ECMO technology continues to evolve, the indications and contraindications for its use also undergo modifications. It remains uncertain whether craniocerebral trauma or cerebral hemorrhage can be classified as an absolute contraindication to ECMO, along with short life expectancy, severe liver disease, acute brain injury, vascular disease, and immunosuppression, as stated in the 2021 ELSO guidelines (11). The use of ECMO in patients with traumatic brain injuries is relatively rare. Parker et al. have retrospectively analyzed 13 adult patients with traumatic brain injury (TBI) who received venovenous ECMO. The results showed that five patients (38.4%) survived to discharge and six patients (46%) received systemic anticoagulation while receiving ECMO. CT did not show an increase in intracranial hemorrhage in all patients, and there were two cases of bleeding complications in the anticoagulation group, both of which were not related to the traumatic brain injury (12). Frickey et al. have reported the successful treatment of a patient with PE after epidural hematoma following VA-ECMO (13). In 2020, Wang conducted a systematic review of 548 patients who received ECMO treatment because of severe trauma. 128 of those patients had traumatic brain injury, 60% of whom initially received heparin anticoagulant therapy (14).

ECMO has gradually been accepted for patients with craniocerebral injuries. The safety and effectiveness of ECMO in patients with craniocerebral injury, as along with the optimal duration of ECMO and anticoagulation strategies, require further study.

## Data Availability

The raw data supporting the conclusions of this article will be made available by the authors, without undue reservation.
